# Structural insights into the interaction of the ribosomal P stalk protein P2 with a type II ribosome-inactivating protein ricin

**DOI:** 10.1038/srep37803

**Published:** 2016-11-25

**Authors:** Xiaojiao Fan, Yuwei Zhu, Chongyuan Wang, Liwen Niu, Maikun Teng, Xu Li

**Affiliations:** 1 Hefei National Laboratory for Physical Sciences at Microscale, Innovation Center for Cell Signaling Networks, School of Life Science, University of Science and Technology of China, Hefei, Anhui, 230026, People’s Republic of China; 2 Key Laboratory of Structural Biology, Hefei Science Center of Chinese Academy of Science, Hefei, Anhui, 230026, People’s Republic of China

## Abstract

Ricin is a type II ribosome-inactivating protein (RIP) that depurinates A^4324^ at the sarcin-ricin loop of 28 S ribosomal RNA (rRNA), thus inactivating the ribosome by preventing elongation factors from binding to the GTPase activation centre. Recent studies have disclosed that the conserved C-terminal domain (CTD) of eukaryotic ribosomal P stalk proteins is involved in the process that RIPs target ribosome. However, the details of the molecular interaction between ricin and P stalk proteins remain unknown. Here, we report the structure of ricin-A chain (RTA) in a complex with the CTD of the human ribosomal protein P2. The structure shows that the Phe^111^, Leu^113^ and Phe^114^ residues of P2 insert into a hydrophobic pocket formed by the Tyr^183^, Arg^235^, Phe^240^ and Ile^251^ residues of RTA, while Asp^115^ of P2 forms hydrogen bonds with Arg^235^ of RTA. The key residues in RTA and P2 for complex formation were mutated, and their importance was determined by pull-down assays. The results from cell-free translation assays further confirmed that the interaction with P stalk proteins is essential for the inhibition of protein synthesis by RTA. Taken together, our results provide a structural basis that will improve our understanding of the process by which ricin targets the ribosome, which will benefit the development of effective small-molecule inhibitors for use as therapeutic agents.

Ricin is a member of the type II ribosome-inactivating protein (RIP) family and has been isolated from the seeds of *Ricinus communis*^1^. This protein is a potential biological weapon, but can also be harnessed for use in biomedicine and has the potential to be used in the treatment of tumours and HIV-infected cells^2^. Ricin consists of a toxic subunit, known as the A chain (RTA), and a subunit that binds galactose and *N*-acetylgalactosamine, known as the B chain (RTB)^3^. RTA has a molecular mass of 30 kDa and exhibits RNA *N*-glycosidase activity, which inactivates ribosomes by removing the A^4324^ in the sarcin-ricin loop of 28 S rRNA^4^. This ribosomal modification prevents GTP hydrolysis by EF-G and subsequently leads to the arrest of protein synthesis at the translocation step^5^. RTA is connected through a disulfide linkage to RTB, which functions in binding to the glycoproteins or glycolipids at the target cell surface and facilitates uptake of the toxin into cells^6^.

Although the sarcin-ricin loop is the direct substrate of RTA, ribosomal proteins have been found to be essential for the depurination process. For example, RTA depurinates the rat ribosome with a *k*_cat_ that is nearly 10^5^-fold greater than that assayed using naked 28 S rRNA^7^. Eukaryotic ribosomal P stalk proteins have been proven to be essential for RTA binding and rRNA depurination. RTA can bind directly to the P1 and P2 proteins of the ribosomal stalk in *Saccharomyces cerevisiae*^8^. Yeast cells encoding mutant ribosomal P stalk proteins exhibit higher *in vivo* resistance to the cytotoxicity of RTA than those encoding wild-type ribosomal P stalk proteins^8^. Other studies have revealed that the depurination activity of RTA in human cells also relies on ribosomal P stalk proteins^9^. A clear binding affinity is observed when RTA is immobilised on a carboxymethylated dextran chip (CM5) and a recombinant human P1-P2 heterodimer is passed over the surface^9^. Ribosomes from P1/P2-depleted cells exhibit a reduced ability to bind to RTA, and the depurination activity of RTA decreases sharply when P1/P2 levels are reduced in human cells^9^.

In humans, the ribosomal P stalk consists of P0, P1 and P2 proteins that form a pentameric complex i.e., P0(P1)_2_(P2)_2_^10^. The N-terminal domain of P0 directly binds to the rRNA with the C-terminal spine helix protruding out. Two P1-P2 heterodimers bind to the spine helix of P0 via the N-terminal domains, whereas the C-terminal tails of P1-P2 heterodimers are unstructured and extend up to 125 Å away from the N-terminal domains^11^,^12^,^13^. A distinctive feature of P stalk proteins is that they contain a highly conserved set of 11 residues (SDDDMGFGLFD) at the C-terminal tail of P2 that recruits elongation factors and interacts with several RIPs, such as trichosanthin and Shiga toxin 1^14^,^15^. The crystal structure of trichosanthin in a complex with the peptide SDDDMGFGLFD (known as C11-P) has been reported previously and revealed that both the N- and C-terminal regions of C11-P are involved in the interaction with trichosanthin^16^.

In this study, we demonstrate that RTA interacts with the human ribosomal P stalk protein P2 with a binding affinity of 4.51 μM and mainly recognises the highly conserved C-terminal tail of P2 (residues 106–115). The structure of RTA in a complex with the peptide DDDMGFGLFD (known as C10-P2), which corresponds to P2 residues 106–115, is determined. Residues on RTA and P2 that are important for complex formation were further confirmed by binding and activity assays. Our results suggest that the conserved DDDM motif of P2 is not involved in the interaction with RTA, but that two consecutive residues, Leu and Phe, in the conserved C-terminal tail of ribosomal P stalk proteins are critical for the interaction with RTA. Thus, small-molecule inhibitors that mimic the 3D conformation of these two consecutive residues deserve further consideration for clinical use in the therapy of ricin poisoning.

## Results

### The structure of RTA in a complex with the C10-P2 peptide

**Figure 1:**
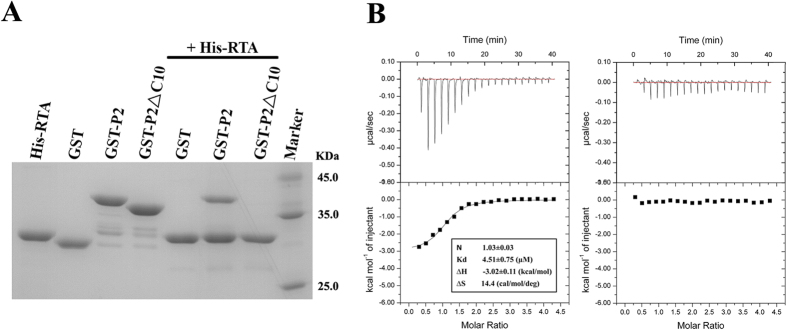
RTA interacts with the conserved C-terminal tail of P2. (**A**) Pull-down analysis of the interaction between RTA and the C-terminal tail of P2. His-RTA bound to wild-type GST-P2 *in vitro*. GST-P2ΔC10 completely lost its ability to associate with His-RTA. His-RTA, GST, GST-P2 and GST-P2ΔC10 were used as controls. (**B**) ITC-based measurements of the binding affinity between RTA and wild-type P2 (left) and between RTA and P2ΔC10 (right).

**Figure 2:**
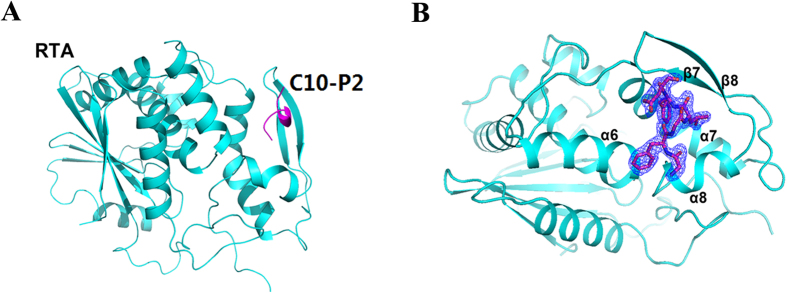
Overall structure of RTA in a complex with the C10-P2 peptide. (**A**) A cartoon representation of the structure of RTA (cyan) in a complex with the C10-P2 peptide (magenta). (**B**) Details of the interaction between RTA and the C10-P2 peptide. The α6, α7, α8, β7 and β8 of RTA form a pocket that recognises the C-terminus of the C10-P2 peptide. Residues in the C-terminus of the C10-P2 peptide are shown as sticks. The 2Fo-Fc electron density map (contoured at 1σ) for the C10-P2 peptide is shown in blue.

Previous research has suggested that RTA binds to the human ribosomal stalk protein P2 both *in vitro* and *in vivo*^9^. To investigate whether the conserved CTD of P2 plays a key role in the interaction with RTA, we constructed a P2 mutant by deleting the 10 C-terminal residues (P2ΔC10) and measured the interaction between P2ΔC10 and RTA through pull-down and isothermal titration calorimetry (ITC) experiments. The results showed that RTA interacts with the wild-type P2 protein with a binding affinity of 4.51 μM but fails to bind to P2ΔC10, indicating that P2 interacts with RTA mainly through the 10 conserved C-terminal residues (Fig. 1A,B). To further elucidate the detailed molecular interaction between RTA and P2, we carried out rigorous trials that aimed to crystallise RTA in a complex with a synthetic C10-P2 peptide. However, we were not able to obtain crystals of the complex. To promote a more efficient interaction between RTA and the synthetic C10-P2 peptide, we constructed a chimeric protein in which C10-P2 was fused to the C-terminus of RTA using four consecutive Gly-Ser (GS) repeats as a linker. This engineered construct proved effective, and diffraction-quality crystals were easily obtained. Finally, we determined the structure of the RTA-C10-P2 fusion protein at a resolution of 1.5 Å. The final model contains one molecule of RTA, one molecule of C10-P2, and 248 water molecules. RTA contains residues 5–263, which display a fold similar to that observed in a previously described structure (PDB: 2AAI). For the C10-P2 peptide, only 6 residues (GFGLFD) could be fitted to the observed electron density; these residues corresponded to Gly^110^-Asp^115^ of the human P2 protein. The 4 N-terminal residues (DDDM) were disordered and did not seem to contribute to RTA binding. As shown in Fig. 2 (A,B), the C-terminus of C10-P2 is coiled into a short helix that binds in a pocket formed by α6, α7, α8, β7 and β8 of RTA. The interface provides a solvent-accessible surface area of 820 Å^2^. The root mean square deviation (RMSD) of the overall main chain of the RTA moiety in RTA-C10-P2 and wild-type RTA is 0.290 Å for 222 comparable C_α_ atoms, indicating that the binding of C10-P2 does not substantially affect the structure of RTA (Supplementary Fig. S1B).

### Molecular interaction between RTA and the C10-P2 peptide

**Figure 3:**
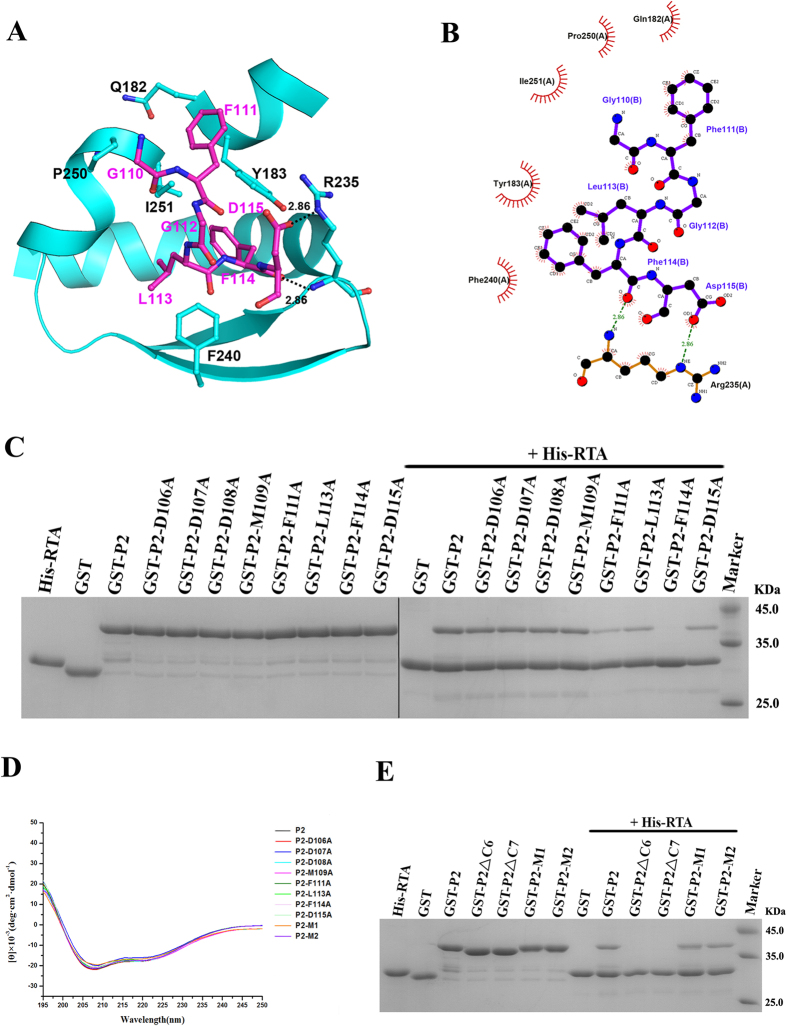
Validation of the residues in the C10-P2 peptide that are responsible for the interaction with RTA. (**A**) A cartoon representation of the binding interface between RTA (cyan) and the C10-P2 peptide (magenta); residues involved in the interaction are labelled and shown as sticks, and hydrogen bonds are indicated by dashed lines. (**B**) Ligplot analysis of the interaction between RTA and the C10-P2 peptide. (**C**) Interaction between RTA and the P2 variants as assessed by pull-down assay. His-RTA, GST, GST-P2, and GST-P2 variants were used as controls. GST-P2-F111A, GST-P2-L113A and GST-P2-D115A showed decreased binding with His-RTA. GST-P2-F114A completely lost the ability to associate with His-RTA. (**D**) CD spectra of recombinant P2 and the P2 mutants. (**E**) The interaction between RTA and the conserved DDDM motif of C10-P2, as assessed by pull-down assay. His-RTA, GST, GST-P2, GST-P2ΔC6 (in which the 6 C-terminal residues were deleted), GST-P2ΔC7 (in which the 7 C-terminal residues were deleted), GST-P2-M1 (in which residues DDD were mutated to AAA) and GST-P2-M2 (in which residues DDDM were mutated to AAAA) were used as controls.

As shown in Fig. 3 (A,B), RTA mainly recognises the C-terminus of C10-P2. The Gly^110^ and Gly^112^ residues of P2 accommodate the required backbone dihedral angles to facilitate the insertion of Phe^111^, Leu^113^ and Phe^114^ into a hydrophobic pocket formed by the Tyr^183^, Arg^235^, Phe^240^ and Ile^251^ residues of RTA (Fig. 3A). The main-chain carbonyl of Phe^114^ in P2 forms a hydrogen bond with the main-chain amide of Arg^235^ in RTA, and the side-chain atom OD1 of Asp^115^ in P2 forms another hydrogen bond with the side-chain atom NE of Arg^235^ in RTA (Fig. 3A, B). Due to their flexibility, the 4 N-terminal residues (DDDM) of C10-P2 could not be traced in the final model. We speculate that these 4 residues may not be involved in the interaction with RTA or that they may play a very weak role. To further evaluate the different roles of the conserved C-terminal residues of P2 in the interaction with RTA and to confirm our structural observations, we constructed eight P2 mutants: P2-D106A (in which residue Asp^106^ was mutated to Ala), P2-D107A (in which residue Asp^107^ was mutated to Ala), P2-D108A (in which residue Asp^108^ was mutated to Ala), P2-M109A (in which residue Met^109^ was mutated to Ala), P2-F111A (in which residue Phe^111^ was mutated to Ala), P2-L113A (in which residue Leu^113^ was mutated to Ala), P2-F114A (in which residue Phe^114^ was mutated to Ala), and P2-D115A (in which residue Asp^115^ was mutated to Ala). We then examined the effects of these mutations on the interaction between P2 and RTA using pull-down assays. As shown in Fig. 3C, the mutants P2-D106A, P2-D107A, P2-D108A and P2-M109A displayed binding affinities for RTA that were similar to that of wild-type P2. In contrast, the mutant P2-F114A did not interact with RTA. The mutants P2-F111A, P2-L113A and P2-D115A exhibited significantly reduced binding to RTA. To ascertain that the binding affinity of these mutant proteins was not due to abnormal folding behaviour, circular dichroism (CD) spectroscopy was performed to examine their secondary structures (Fig. 3D). The secondary structures of these mutant proteins were similar to that of wild-type P2, suggesting that none of the above mutations appeared to affect overall protein structure during expression of the mutant recombinant proteins. These results indicated that the binding of P2 and RTA is mainly mediated by hydrophobic interactions and that the Phe^111^, Leu^113^ and Phe^114^ residues of P2 are critical for the RTA-P2 interaction. The hydrogen bond formed by Asp^115^ in P2 and Arg^235^ in RTA is also important for P2-mediated ribosomal anchoring of RTA. In addition, the 4 N-terminal residues (DDDM) of C10-P2 contribute little to RTA binding, and this finding is consistent with our structural observations.

### The conserved DDDM motif of C10-P2 contributes little to the interaction with RTA

As the DDDM motif of C10-P2 could not be traced in the final structural model and the pull-down assays showed that the single point mutations of these residues did not change the binding affinity for RTA, it is likely that the conserved DDDM motif has no effect on the interaction between C10-P2 and RTA. To test this hypothesis, we constructed four P2 mutants — P2ΔC6 (in which the 6 C-terminal residues were deleted), P2ΔC7 (in which the 7 C-terminal residues were deleted), P2-M1 (in which the residues DDD were mutated to AAA), and P2-M2 (in which the residues DDDM were mutated to AAAA) — and measured their interaction with RTA using pull-down assays. As shown in Fig. 3E, P2ΔC6 and P2ΔC7 did not interact with RTA, and P2-M1 and P2-M2 had an RTA binding affinity that was similar to that of wild-type P2. The mutations in P2-M1 and P2-M2 did not appear to affect the overall protein structure, as shown by CD analysis (Fig. 3D). These results suggest that the conserved DDDM motif at the C-terminus of P2 is not involved in the interaction with RTA, which is consistent with our structural observations.

### Perturbation of the RTA-P2 interaction affects the ability of RTA to inhibit protein synthesis

**Figure 4:**
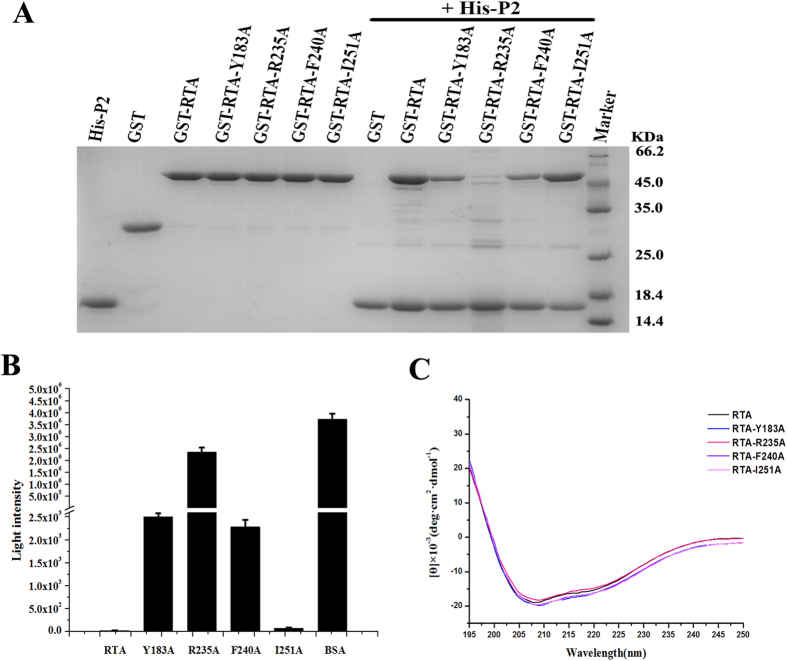
Disruption of the interaction between RTA and P2 affects the ability of RTA to inhibit protein synthesis. (**A**) The interactions between RTA variants and P2, as assessed by pull-down assay. GST-RTA-Y183A and GST-RTA-F240A showed decreased binding with P2. GST-RTA-R235A almost completely lost the ability to associate with His-P2. His-P2, GST-RTA, and GST-RTA variants were used as controls. (**B**) The ability of RTA to inhibit protein synthesis was assayed using an *in vitro* cell-free translation experiment. Rabbit reticulocyte lysates (Promega) were used as the source of ribosomes. Protein translation efficiency was determined by measuring the activity of luciferase using a Veritas^TM^ Microplate Luminometer (Veritas). BSA was used as a control. (**C**) CD spectra of the recombinant RTA and RTA mutants.

Previous results have suggested that the Phe^111^, Leu^113^, Phe^114^ and Asp^115^ residues of P2 are responsible for the interaction with RTA. To further confirm our structural observations and validate whether the residues of RTA that make close contact with these 4 residues are also critical for the interaction with P2, we made four RTA mutants — RTA-Y183A (in which residue Tyr^183^ was mutated to Ala), RTA-R235A (in which residue Arg^235^ was mutated to Ala), RTA-F240A (in which residue Phe^240^ was mutated to Ala) and RTA-I251A (in which residue Ile^251^ was mutated to Ala) — and performed pull-down assays to examine the effects of these mutations on the formation of the RTA-P2 complex. As shown in Fig. 4A, the RTA-I251A mutant had a P2 binding affinity that was similar to that of wild-type RTA. By contrast, the mutations RTA-Y183A and RTA-F240A greatly decreased the binding affinity of P2 for RTA, and the RTA-R235A mutation nearly completely abolished the interaction between P2 and RTA. We then investigated the effects of these mutations on the ability of RTA to inhibit protein synthesis using a cell-free translation system. We used rabbit reticulocyte lysates as the source of ribosomes. In the absence of RTA, ribosomes translated mRNA encoding luciferase normally and generated a high luciferase signal. When mixed with RTA, only a very low luciferase signal was detected owing to efficient inhibition of protein synthesis by RTA. As shown in Fig. 4B, the luciferase signals detected in the presence of each of the RTA mutants were increased compared with that detected in the presence of wild-type RTA. The luciferase signal differed among the mutants, which are listed here in order of decreasing luciferase signal: RTA-R235A, RTA-Y183A, RTA-F240A, and RTA-I251A. CD analysis showed that these assayed mutant proteins were correctly folded (Fig. 4E). These results from the cell-free translation assay are consistent with the previous pull-down assay data, further confirming our structural observations.

## Discussion

We constructed a chimeric protein in which C10-P2 was fused to the C-terminus of RTA via a GSGSGSGS linker and determined the structure of the resulting RTA-C10-P2 fusion protein. To elucidate whether the linker affected the formation of the RTA-C10-P2 complex, we analysed the flexibility and length of the linker, the electron density for linker to the peptide, and the effects of crystal packing. It has been reported that flexible Gly- and Ser-rich linkers can form loops without imposing any constraints on the conformation or interactions of the linked partners^17^. This notion is consistent with the result that no electron density was observed in our structural model for the GSGSGSGS linker to the peptide. In our structural model, 16 residues are missing between Pro^263^ of RTA and Gly^110^ of P2, and the distance between these two residues is approximately 29 Å. When these 16 residues form a β-strand, the length of this strand is predicted to be 56 Å, which is much longer than 29 Å. Based on the above considerations, we believe that the linker may adopt a flexible conformation of sufficient length and thus does not disturb the interaction between the P2 peptide and RTA. Moreover, we also paid attention to crystal packing, which could disturb the interaction between the P2 peptide and RTA; however, no steric effect from the partner or symmetry-related molecule was observed. Therefore, we inferred that the binding of the P2 peptide to RTA is not due to a steric effect but results from its physiological binding status.

Our structural model and the pull-down assay results showed that the conserved DDDM motif of C10-P2 has little effect on the interaction with RTA and that the binding between C10-P2 and RTA is mediated by hydrophobic interactions (i.e., the Phe^111^, Leu^113^ and Phe^114^ residues of P2 inserted into a hydrophobic pocket formed by the Tyr^183^, Arg^235^, Phe^240^ and Ile^251^ residues of RTA) and hydrogen bonds (i.e., the Phe^114^ and Asp^115^ residues of P2 form hydrogen bonds with the Arg^235^ residue of RTA). Ito *et al*. reported the structure of a complex of the CTD of the archaeal stalk protein aP1 and GDP-bound aEF1α, and confirmed that the structure was stabilised by many hydrophobic interactions^18^. They also inferred that eukaryotic P1/P2 interacts directly with eEF1α via hydrophobic interactions. Our structural model and pull-down assay results show that RTA interacts with C10-P2 via many hydrophobic interactions, which is similar to the interaction between eEF1α and P1/P2 (CTD). By contrast, previous studies have shown that in *S. cerevisiae*, several arginines of RTA are involved in the interaction between RTA and the ribosome. Li *et al*. found that seven single mutations (R189A, R191A, R193A, R196A, R197A, R234A, and R235A) did not reduce the toxicity of RTA compared with wild-type RTA, whereas three double mutations (R193A-R235A; R189A-R234A; and R191A-R196A) did reduce the toxicity of RTA compared with wild-type RTA. They also reported that the R193A-R235A mutant showed the greatest reduction in RTA toxicity in *S. cerevisiae*. In this paper, the authors analysed the correlation between doubling times and viability; wild-type RTA (8.1 ± 1.3 h), R189A-R234A (8.0 ± 1.1 h), and R191A-R196A (8.2 ± 1.0 h) exhibited very similar doubling times, which shows that Arg^189^, Arg^191^, Arg^196^ and Arg^234^ are not important for RTA toxicity in *S. cerevisiae*^19^. Consistent with the above-mentioned results, our pull-down assay results show that the single mutation R235A nearly completely abolished the interaction between the human stalk protein P2 and RTA; however, other arginines (Arg^189^, Arg^191^, Arg^193^, Arg^196^, and Arg^234^) did not interact with the P2 peptide in our structural model. Together with other reports, our structural and functional studies indicate that hydrophobic interactions and hydrogen bonds contribute to the interaction between C10-P2 and RTA.

Hydrophobic forces dominate the interaction between RTA and P2. Two consecutive hydrophobic residues, Leu and Phe, in the conserved C-terminal tail of P2 are important for the interaction between P2 and RTA. The crystal structure of trichosanthin in a complex with the peptide SDDDMGFGLFD (C11-P) has been reported previously^16^. By comparing the structure of this complex with that of the RTA-P2 complex, we found that although RTA and trichosanthin adopt a similar fold (having an RMSD of 0.786 Å for 189 comparable Cα atoms) (Supplementary Fig. S1C), the conformation of the C10-P2 peptide bound to RTA differs significantly from the conformation of C11-P bound to trichosanthin. However, surprisinglyly, trichosanthin also recognises the consecutive Leu and Phe residues in the C11-P peptide, which inserts into a pocket that is formed by the Phe^166^, Ala^218^, Val^223^ and Asn^236^ residues of trichosanthin. Moreover, sequence alignment suggests that the LF motif is the unique element that is conserved in both eukaryotic and archaeal ribosomal stalk proteins; this motif has been shown to be necessary for the binding of translation factors to ribosomal stalk proteins (Supplementary Fig. S2)^18^,^20^. These results indicate that RTA, trichosanthin and translation factors all interact with the highly conserved LF motifs by forming hydrophobic interactions.

**Figure 5:**
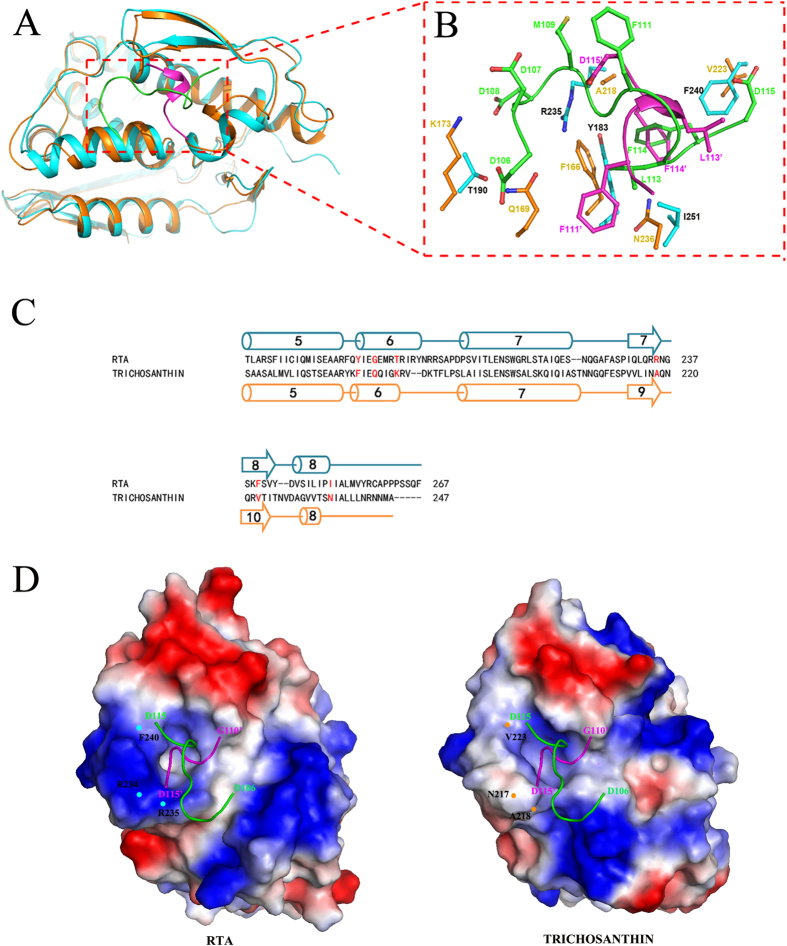
Differences between the C10-P2 recognition models of RTA and trichosanthin. (**A**) Structural superpositions of RTA-C10-P2 (in which C10-P2 (magenta) was covalently linked to RTA (cyan)) and trichosanthin-C11-P2 (in which the C11-P2 (green) peptide was bound to trichosanthin (orange)). The residues involved in the interaction are shown as sticks, with colours representing atom identity. (**B**) An enlarged view of the superposed C10-P2 peptide interface of RTA and trichosanthin. Residues at the interface are labelled and highlighted in colour. (**C**) Sequence alignment of the C-terminal domains of RTA and trichosanthin. The secondary structural elements of the C-terminal domains of RTA and trichosanthin are shown above and below the sequences, respectively. Residues of RTA and trichosanthin that are involved in the interaction with C10-P2 are labelled and shown in red. (**D**) A comparison of the hydrophobic pockets of RTA and trichosanthin when bound to either C10-P2 or C11-P. Red and blue surfaces represent negative and positive electrostatic potentials, respectively. Important residues of RTA and trichosanthin are highlighted in cyan and orange, respectively. The C10-P2 and C11-P peptides are shown in magenta and green, respectively.

Despite their similarity, significant differences remain between the P stalk protein recognition models of RTA and trichosanthin. In the structure of the trichosanthin-C11-P complex, the Asp^108^ residue of C11-P interacts with the Lys^173^ residue of trichosanthin via salt bridges, while the Asp^106^ residue of C11-P forms hydrogen bonds with the Gln^169^ residue of trichosanthin (Fig. 5A,B). The substitution of these two aspartate residues in the P2 protein with alanine abolishes the interaction between trichosanthin and P2^16^. However, the corresponding aspartate residues of P2 are disordered in the RTA-C10-P2 complex, and our pull-down assay results indicate that these residues do not affect the interaction between RTA and P2. This finding is also consistent with the structural features of RTA. The Gly^186^ and Thr^190^ residues in RTA, which correspond to Gln^169^ and Lys^173^ in trichosanthin, lose the ability to form hydrogen bond and salt-bridge interactions with the Asp^106^ and Asp^108^ residues of P2 (Fig. 5B,C). Moreover, Arg^235^ of RTA, which plays a vital role in the interaction with C10-P2, results in steric hindrance with the N-terminus of the C11-P peptide (Fig. 5D). In trichosanthin, the corresponding residue is Ala^218^, which substantially aids the binding of the N-terminus of the C11-P peptide to trichosanthin (Fig. 5D). Figure 5D also shows steric hindrance that is due to the Asp115 (D115’) of C10-P2, which affects binding to trichosanthin at Asn^217^ (N217), and Asp115 (D115) of C11-P, which affects binding to RTA at Phe^240^ (F240).

Based on all of our findings, we speculate that two consecutive residues, Leu and Phe, in the conserved C-terminal tail of ribosomal P stalk proteins may be the smallest critical elements for anchoring RIPs, such as RTA and trichosanthin. Molecular simulation of the conserved C-terminal tail of ribosomal P stalk proteins will be a useful tool for the development of novel small-molecule drugs for use in treating RIP poisoning. In addition, our results provide a P stalk protein recognition model that is distinct from that of the type I RIP trichosanthin, suggesting that although the region of RIPs that binds different ribosomal P stalk proteins may be similar, the respective molecular recognition models might significantly differ in their details.

## Methods

### Cloning, expression and purification

The gene encoding RTA was a kind gift from the Second Military Medical University. The fusion gene encoding RTA (1–267)-linker-C10-P2 (106–115) was constructed by PCR and cloned into a modified pET-28a (+) vector with a 6×His tag using the NcoI/XhoI restriction site. Overexpression of the chimeric protein was induced in *Escherichia coli* C43 (DE3) (Novagen) by adding 0.5 mM isopropyl-β-D-thiogalactoside (IPTG) when the cell density reached an OD_600 nm_ of 0.6–0.8. After culturing for approximately 20 h at 289 K, the cells were collected and lysed. The recombinant protein was purified using Ni^2+^-nitrilotriacetate affinity resin (Ni-NTA, Qiagen) in buffer A (50 mM Tris-HCl pH 7.5, 200 mM NaCl and 5% glycerol) and was eluted with 200 mM imidazole. The chimeric protein was further purified using HiLoad 16/60 Superdex 200 (GE Healthcare) in buffer B (50 mM Tris-HCl pH 7.5 and 100 mM NaCl) and was concentrated to 9 mg/ml for use in crystallisation trials.

For functional studies, DNA fragments of RTA (1–267) were inserted into the modified pET-28a (+) vector and the pGEX-6P-1 vector. The P2 gene was amplified by PCR from a human cDNA library using PrimeSTAR HS DNA polymerase (TaKaRa) and was cloned into the modified pET-28a (+) vector and the pGEX-6P-1 vector. The recombinant His-RTA proteins were expressed and purified in buffer C (50 mM sodium phosphate buffer pH 7.5 and 5% glycerol) using the same procedure as that used for the chimeric protein. His-P2 protein was expressed in *E. coli* BL21 (DE3) (Novagen) and purified under identical conditions to those used for recombinant His-RTA. GST and the recombinant GST fusion proteins were purified in buffer C using glutathione-affinity resin (GE Healthcare) and were eluted with 20 mM reduced glutathione. All of the mutants in this study were generated using the MutanBEST kit (TaKaRa), and were expressed and purified in the same manner as that used for the wild-type proteins.

### Crystallisation and Diffraction Data Collection

Crystals of the chimeric protein were grown using the hanging-drop vapour-diffusion method at 289 K and yielded crystals in two days when using a well solution that contained 0.4 M ammonium dihydrogen phosphate. For data collection, all crystals were soaked in a cryoprotectant solution consisting of the respective reservoir solution supplemented with 20% (v/v) glycerol, and they were then flash-frozen in liquid nitrogen. Data sets for all crystals were collected on beamline 17U at the Shanghai Synchrotron Radiation Facility (SSRF) at 100 K. The data were processed and scaled using the *HKL2000*^21^ package and programmes in the *CCP4* package^22^. Statistical parameters for the diffraction data are summarised in Table 1.

### Structure determination and refinement

The structure of the chimeric protein was determined by molecular replacement using *MOLREP*^23^, as implemented in the *CCP4* suite^22^. The structure of ricin A-chain (Protein Data Bank code 2AAI) was used as the search model. An initial model containing the ricin A-chain was obtained and refined using the maximum likelihood method, as implemented in *REFMAC5*^24^. The C10-P2 peptide was manually rebuilt in *Coot*^25^. The final model was evaluated using the programmes *MOLPROBITY*^26^ and *PROCHECK *27. Sequence alignment was performed using the programme *ClustalW *28. The crystallographic parameters are listed in Table 1. All of the structures shown in the figures were prepared using *PyMOL* (DeLano Scientific).

### Pull-down assays

Similar amounts of purified recombinant His-RTA were immobilised on 100 μl of Ni^2+^-nitrilotriacetate beads for 30 min at 277 K. After washing three times with binding buffer C (1 ml each time), the beads were incubated with 1 ml of purified GST or GST fusion proteins at 277 K for 1 h. The beads were then washed thoroughly in buffer C supplemented with 30 mM imidazole. Finally, the bound proteins were eluted from the beads with 100 μl of buffer C supplemented with 200 mM imidazole. All samples were resolved by SDS-PAGE (15%) and stained with Coomassie Brilliant Blue. Samples of 3.6 μg His-RTA, 0.7 μg GST, 1.4 μg GST-P2 and similar amounts of the GST-P2 mutants were used as controls. Pull-down assays assessing the interactions of GST-RTA or the GST-RTA mutants with purified His-P2 were performed in a similar way to that described above. Samples of 3.4 μg His-P2, 0.7 μg GST, 1.5 μg GST-RTA and similar amounts of the GST-RTA mutants were used as controls.

### Isothermal titration calorimetry (ITC)

ITC measurements were performed using an ITC-200 titration calorimeter (GE Healthcare). The measurements were performed at 298 K in buffer C. Human ribosomal P2 protein or P2ΔC10 protein at a concentration of 1 mM was titrated into wild-type RTA (50 μM). Control experiments were performed under identical conditions by titrating P2 into buffer alone or buffer into RTA alone. The ITC data were subsequently fitted to a single-site model using Origin 7.0 (OriginLab); the model was provided by the manufacturer.

### Cell-free translation inhibition experiment

The enzymatic activity of wild-type RTA and the RTA variants was measured in a cell-free *in vitro* translation system using rabbit reticulocyte lysates (Promega) as the source of ribosomes. The protein translation efficiency was determined by measuring the activity of luciferase. After pre-incubating rabbit reticulocyte lysates with 2 ng RTA or RTA variants, 0.5 μg of luciferase template mRNA was added, followed by incubation for 90 min at 303 K. The synthesis of functional luciferase was assayed in the reaction products using the Luciferase Reporter Assay System (Promega). Then, 100 μl of Luciferase Assay Reagent was added to the wells of a 96-well plate, each of which contained 20 μl of the reaction product. Luciferase activity was measured using a Veritas^TM^ Microplate Luminometer (Veritas). The read time was 1 second, and the number of runs was 3. Bovine serum albumin (BSA), which could not bind to the ribosomes and affect their translation activity, was used as a control.

### Circular dichroism (CD) spectroscopy

The purified P2 and P2 mutants, diluted to 0.15 mg/ml in 50 mM sodium phosphate buffer (pH 7.5), were loaded into a quartz cuvette (d = 0.1 cm path length), and CD spectra were recorded from 190 to 260 nm on a JASCO-J810 spectropolarimeter at 298 K. A buffer-only sample was used as a reference. All CD spectra represent the average of three successively obtained spectra. Molar ellipticities (θ) were plotted against wavelength, and the reference curve was subtracted from each curve. CD spectra of the purified RTA and RTA mutants were performed using the same procedure as that used for P2.

## Additional Information

**Accession codes:** The atomic coordinates and structure factors have been submitted to the Protein Data Bank under the accession code 5DDZ.

**How to cite this article**: Fan, X. *et al*. Structural insights into the interaction of the ribosomal P stalk protein P2 with a type II ribosome-inactivating protein ricin. *Sci. Rep.* **6**, 37803; doi: 10.1038/srep37803 (2016).

**Publisher's note:** Springer Nature remains neutral with regard to jurisdictional claims in published maps and institutional affiliations.

## Supplementary Material

Supplementary Figures

## Figures and Tables

**Table 1:** Data collection and Refinement Statistics for RTA-C10-P2 complex.

Data collection statistics	RTA-C10-P2
Space Group	*P*4_1_2_1_2
Unit Cell Parameters	
*a, b, c* (Å)	67.6, 67.6, 140.7
*α, β, γ* (˚)	90.0, 90.0, 90.0
Wavelength (Å)	0.9792
Resolution limits (Å)	50.00–1.50 (1.55–1.50)^a^
No. of unique reflections	52756
Completeness (%)	99.7 (99.6)
Redundancy	11.9 (8.8)
*R*_merge_ (%)^b^	10.8 (82.5)
*R*_p.i.m_ (%)	3.3 (28.5)
Mean I/σ (I)	26.5 (2.6)
**Refinement Statistics**	
Resolution limits (Å)	50.00–1.50
*R*_work_ (%)^c^/*R*_free_ (%)^d^	14.62/17.52
Rmsd for bonds (Å)	0.009
Rmsd for angles (˚)	1.240
B factor (Å^2^)	
Protein	24.41
Water	41.18
No. of non-hydrogen protein atoms	2071
No. of water oxygen atoms	248
**Ramachandran plot** (**%**)	
Most favored regions	95.7
Additional allowed regions	3.9
Generously allowed regions	0.4

^a^Values in parentheses are for the highest-resolution shell.

^b^*R*_*merge*_ = ∑|I_i_ − <I>|/∑|I|, where I_i_ is the intensity of an individual reflection and <I> is the average intensity of that reflection.

^c^*R*_*work*_ = ∑||F_o_| − |F_c_||/∑|F_o_|, where F_o_ and F_c_ are the observed and calculated structure factors for reflections, respectively.

^d^*R*_*free*_ was calculated as *R*_*work*_ using the 5% of reflections that were selected randomly and omitted from refinement.
