# Supplementary material for: Structural insights into the interaction of the ribosomal P stalk protein P2 with a type II ribosome-inactivating protein ricin
Source: Sci Rep. 2016 Nov 25;6:37803. doi: 10.1038/srep37803 (PMC5122897; doi:10.1038/srep37803)
Supplement: Supplementary Figures [file srep37803-s1.pdf]

Structural insights into the interaction of the ribosomal P stalk protein P2 with a type II ribosome-inactivating protein ricin

Xiaojiao Fan[1,2,+], Yuwei Zhu[1,2, +], Chongyuan Wang [1,2], Liwen Niu[1,2], Maikun Teng[1,2,*] and Xu Li[1,2,*]

[1]Hefei National Laboratory for Physical Sciences at Microscale, Innovation Center for Cell Signaling Networks, School of Life Science, University of Science and Technology of China, Hefei, Anhui, 230026, People's Republic of China

[2]Key Laboratory of Structural Biology, Hefei Science Center of Chinese Academy of Science, Hefei, Anhui, 230026, People's Republic of China

[+]These authors contributed equally to this work.

*Correspondence should be addressed to Xu Li (sachem@ustc.edu.cn) or Maikun Teng (mkteng@ustc.edu.cn).

Synopsis: We determined the structure of ricin-A chain (RTA) in a complex with the C-terminal domain (CTD) of the human ribosomal protein P2. Combining this information with binding and activity assays, we identified the residues on RTA and P2 that are important for complex formation.

Supplementary information

Figure S1 Stereo view of the structure of RTA (RTA-C10-P2 complex) alone and comparison of RTA (PDB: 2AAI) and trichosanthin (PDB: 2JDL). (A) Stereo view of the structure of RTA (RTA-C10-P2 complex). (B) The alignment of RTA (RTA-C10-P2 complex) with RTA (PDB: 2AAI). (C) The alignment of RTA (RTA-C10-P2 complex) with trichosanthin (PDB: 2JDL). RTA (RTA-C10-P2 complex), RTA (PDB: 2AAI) and trichosanthin are shown in cyan, wheat and orange, respectively.

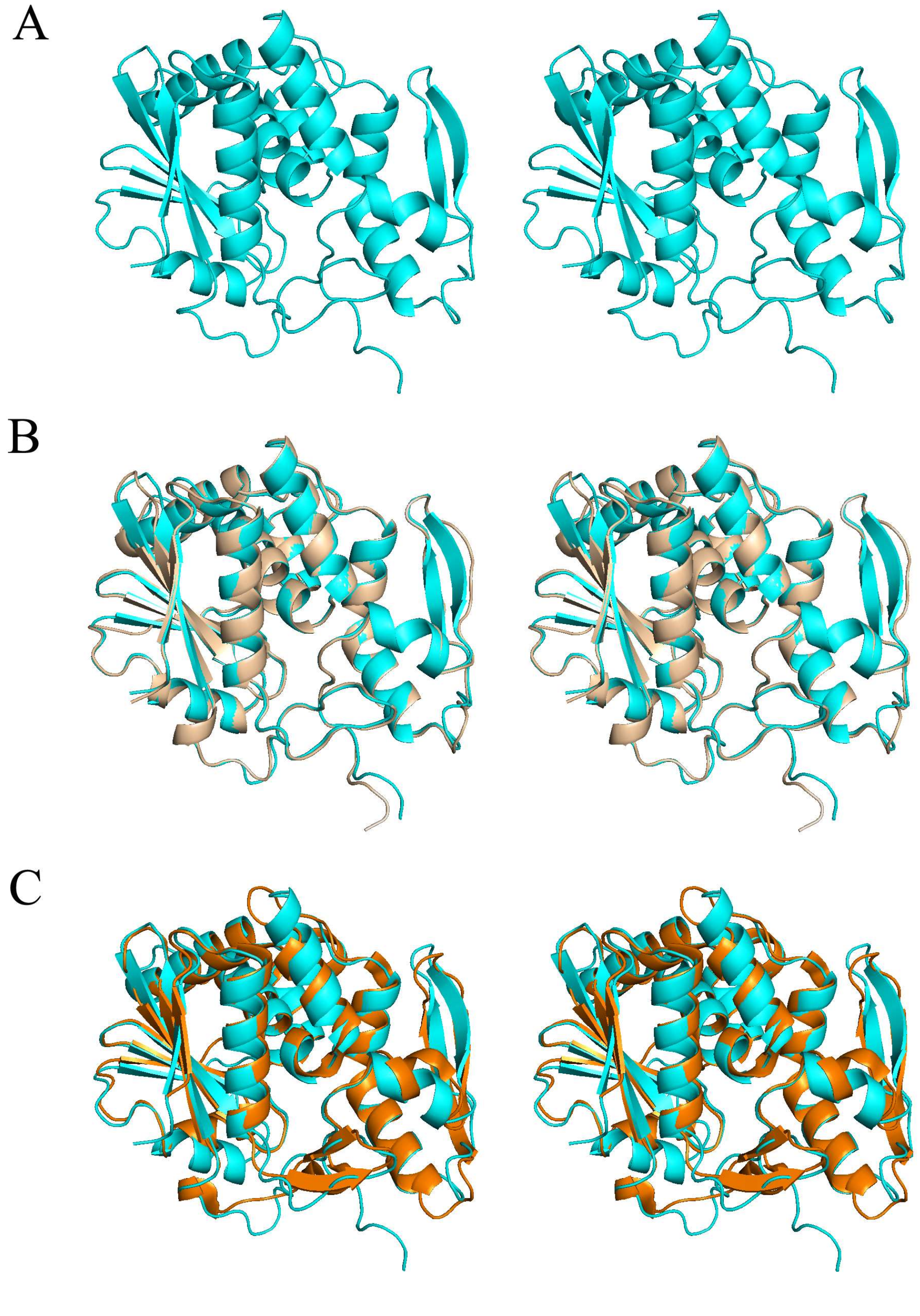

Figure S2 Sequence alignment of the CTDs of various stalk proteins. The sequences for archaeal aP0 (L10) and aP1 (L12) from *Pyrococcus horikoshii* (*Pho*), *Haloarcula marismortui* (*Hma*), *Sulfolobus solfataricus* (*Sso*), *Sulfolobus acidocaldarius* (*Sac*) and *Methanococcus maripaludis* (*Mmp*). The sequences for Eukaryotic P0/P1/P2 from *Saccharomyces cerevisiae* (*Sce*), *Schizosaccharomyces pombe* (*Spo*), *Arabidopsis thaliana* (*Ath*), *Caenorhabditis elegans* (*Cel*), *Drosophila melanogaster* (*Dme*), *Mus musculus* (*Mmu*) and *Homo sapiens* (*Hsa*). Strictly conserved residues and highly conserved residues are depicted with red and green, respectively.

P0_*Pho*	329	-SEEEALAGLSALFG--	342
P1_*Pho*	95	-SEEEALAGLSALFG--	108
P0_*Hma*	335	DDDEDAGDALGAMF---	348
P1_*Hma*	102	-DDEASGEGLGELFG--	115
P0_*Sso*	325	-SEEEIASGLASLFG--	338
P1_*Sso*	93	-SEEEIASGLASLFG—	106
P0_*Sac*	322	--EEEIGGGLSSLFGG-	335
P1_*Sac*	92	-SEEEIGGGLSSLFG--	105
P0_*Mmp*	322	KEEAAAPAGLGMLF---	335
P1_*Mmp*	86	-TGAAAAAGLGALFG--	99
P0_*Sce*	299	-EEESDDDMGFGLFD--	312
P1-A_*Sce*	93	-KEESDDDMGFGLFD--	106
P1-B_*Sce*	93	-AEESDDDMGFGLFD--	106
P2-A_*Sce*	93	-AEESDDDMGFGLFD--	106
P2-B_ *Sce*	97	-KEESDDDMGFGLFD--	110
P0_*Spo*	299	-EEESDEDMGFGLFD--	312
P1-A1_*Spo*	96	-EEESDEDMGFGLFD--	109
P1-A3_*Spo*	97	-EEESDEDMGFGLFD--	110
P2-A_*Spo*	97	-EEESDEDMGFGLFD--	110
P2-B_*Spo*	97	-AEESDEDMGFGLFD--	110
P0-1_*Ath*	304	-AEESDGDMGFDLFG--	317
P0-2_*Ath*	307	---EEDYGGDFGLFDEE	320
P1-1_*Ath*	99	-AEESDGDLGFGLFD--	112
P2-1_*Ath*	102	-KEESDDDMGFSLFE--	115
P2-3_*Ath*	102	-DESSDDAGMMGLFD--	115
P2-5_*Ath*	101	-EESEDDGGMMSLFD--	114
P3-1_*Ath*	106	-SEEEEGDFGFDLFG--	119
P0_*Cel*	299	-KEESDDDMGFGLFD--	312
P1_*Cel*	98	-KEESDDDMGFGLFD--	111
P2_*Cel*	94	-KEESDDDMGFGLFD--	107
P0_*Dme*	304	-SEEEDDDMGFGLFD--	317
P1_*Dme*	99	-SDQSDDDMGFGLFD--	112
P2_*Dme*	100	-SESEDDDMGFALFE--	113
P0_*Mmu*	304	-SEESDEDMGFGLFD--	317
P1_*Mmu*	101	-SEESEDDMGFGLFD--	114
P2_*Mmu*	102	-SEESDDDMGFGLFD--	115
P0_*Hsa*	304	-SEESDEDMGFGLFD--	317
P1_*Hsa*	101	-SEESDDDMGFGLFD--	114
P2_*Hsa*	101	-SEESDDDMGFGLFD--	114
